# A post-mortem population survey on foetal-infantile end-of-life decisions: a research protocol

**DOI:** 10.1186/s12887-018-1218-4

**Published:** 2018-08-03

**Authors:** Laure Dombrecht, Kim Beernaert, Ellen Roets, Kenneth Chambaere, Filip Cools, Linde Goossens, Gunnar Naulaers, Luc De Catte, Joachim Cohen, Luc Deliens, on behalf of the NICU consortium, Sabrina Laroche, Claire Theyskens, Christine Vandeputte, Luc Cornette, Hilde Van de Broek

**Affiliations:** 1 End-of-Life Care Research Group, Ghent University & Vrije Universiteit Brussel (VUB), Ghent, Belgium; 2 Department of Obstetrics, Women’s Clinic, University Hospital Ghent, Ghent, Belgium; 3 Department of Neonatology, Universitair Ziekenhuis Brussel, Vrije Universiteit Brussel, Brussel, Belgium; 4 Department of Neonatology, Ghent University Hospital, Ghent, Belgium; 5 Department of Development and Regeneration, KU Leuven, Leuven, Belgium; 6 Division of Woman and Child, Clinical Department of Obstetrics and Gynecology, Fetal Medicine Unit, University Hospitals Leuven, Leuven, Belgium; 7 Antwerp University Hospital, Antwerp, Belgium; 8 Hospital Oost-Limburg Genk, Genk, Belgium; 9 Hospital GZA St Augustinus, Antwerp, Belgium; 10 AZ St Jan Brugge, Brugge, Belgium; 11 ZNA Middelheim, Antwerp, Belgium

**Keywords:** End-of-life decisions, Neonates, Stillbirths, Termination of pregnancy, Mortality follow-back survey, Population-based

## Abstract

**Background:**

The death of a child before or shortly after birth is frequently preceded by an end-of-life decision (ELD). Population-based studies of incidence and characteristics of ELDs in neonates and infants are rare, and those in the foetal-infantile period (> 22 weeks of gestation – 1 year) including both neonates and stillborns, are non-existent. However, important information is missed when decisions made before birth are overlooked. Our study protocol addresses this knowledge gap.

**Methods:**

First, a new and encompassing framework was constructed to conceptualise ELDs in the foetal-infantile period. Next, a population mortality follow-back survey in Flanders (Belgium) was set up with physicians who certified all death certificates of stillbirths from 22 weeks of gestation onwards, and infants under the age of a year. Two largely similar questionnaires (stillbirths and neonates) were developed, pilot tested and validated, both including questions on ELDs and their preceding decision-making processes. Each death requires a postal questionnaire to be sent to the certifying physician. Anonymity of the child, parents and physician is ensured by a rigorous mailing procedure involving a lawyer as intermediary between death certificate authorities, physicians and researchers. Approval by medical societies, ethics and privacy commissions has been obtained.

**Discussion:**

This research protocol is the first to study ELDs over the entire foetal-infantile period on a population level. Based on representative samples of deaths and stillbirths and applying a trustworthy anonymity procedure, the research protocol can be used in other countries, irrespective of legal frameworks around perinatal end-of-life decision-making.

## Background

Recent decades have seen an increase in possible medical and technical interventions for critically ill neonates and infants [1]. However, in Flanders, Belgium about 8.7 per thousand children still die during the foetal-infantile period, i.e. from foetuses of more than 500 g or 22 weeks of gestation up until 1 year after birth [2]. This is comparable with death rates reported, for instance, in the United States [3]. Many of these deaths occur at neonatal intensive care units (NICUs) and are preceded by a possibly life-shortening end-of-life decision (ELD) [4–6]. In neonates, these include non-treatment decisions such as withholding or withdrawing life-sustaining treatment, intensification of alleviation of pain and/or other symptoms with a potential life-shortening effect and intentionally ending life with lethal drugs [7]. Additionally, prenatal diagnostic techniques (genetic techniques, prenatal imaging techniques) have evolved considerably, leading to an increasing number of congenital malformations being diagnosed prenatally instead of after birth [8, 9]. Some decisions such as abstinence from treatment [8–10] or termination of pregnancy (TOP) [8, 9] can be made during gestation in cases of the detection of serious abnormalities [11–13]. For stillbirths from 22 weeks of gestation and onwards – which is considered as the definition of a viable foetus by the WHO – TOPs are considered late terminations. Stillborns and deceased neonates cannot be seen as separate patient populations, since they are in essence the same patient where an ELD can be made either before or after birth. The only difference is therefore the occurrence of birth and not necessarily a difference in disorders or congenital anomalies. Research into end-of-life decision-making on a population level should therefore take into account the foetal-infantile period in its entirety (instead of both periods separately). This is needed to provide reliable incidence rates and information on the decision-making process in this vulnerable population. Evaluation and monitoring of ELD practice in the entire foetal-infantile period could lead to better understanding of current prenatal and neonatal health care and detect points of improvement since there have been no all-inclusive guidelines up to the present.

Population-based studies (i.e. with *all* death cases as the focus) are ideal to study the incidence and characteristics of ELDs, but such studies are rare in neonates and infants [14–16] and, to our knowledge, non-existent in stillborns. In neonates, results are mostly based on reviews of medical records of a NICU at a particular hospital. In these studies 40 to 93% of deaths in a NICU follow withdrawal of life-sustaining treatments [6, 17–19]. The larger scale EURONIC study was based on physicians’ self-reported practices within 143 European NICUs in the 1990s [20]. The only population-based studies are from the Netherlands (in 2014) [15] and Belgium (in 2000) [14]. These studies found an ELD being made in 60% of all deaths of neonates and infants. In stillborns, previous studies in 2003 [11] and in 2000–2005 [13] have only looked at the prevalence of late TOP [11, 13, 21]. Not much is known about the entirety of end-of-life practices (including decisions other than TOP) and their decision-making process, or about patient characteristics besides gestational age and the presence of foetal anomalies.

We developed a study design to evaluate and monitor ELDs and their decision-making process across the entire foetal-infantile period in Flanders, Belgium. The study design involves the development of a validated conceptual framework of ELDs spanning the entire foetal-infantile period (based on existing frameworks) and the development of a survey methodology that addresses the particular difficulties in capturing and surveying stillbirths and neonatal deaths, and provides opportunities for comparison of ELD practices between hospitals.

## Methods

This population study has the design of a mortality follow-back survey based on all death certificates of stillbirths and neonates. Questionnaires are either sent to the certifying physicians by post or are provided at maternity wards. In order to develop these questionnaires, adjustments to an existing neonatal ELD framework needed to be made.

### Conceptual framework of foetal-infantile ELDs

Prenatal ELDs should be taken into account when presenting a reliable and complete picture on foetal-infantile ELD practices. However, to date these prenatal ELDs have not been included in a comprehensive framework with neonatal ELDs. We adjusted a previously existing and validated framework of ELDs in neonates [7] in order to include both prenatal and neonatal ELDs. This framework [7] includes three dimensions: ‘medico-technical‘, ‘medico-ethical’ and ‘consultation with parents’. The dimension ‘consultation with parents’ was excluded from our own framework since no decision can be made prenatally without at least the mother consenting to an intervention. Furthermore, the dimension ‘consultation with patients’ is also excluded from the adult ELD framework where the medical decision and its intention are the only determinants of an ELD. However, this dimension is still very important which is why consultation with parents will still be addressed in detail by means of additional questions outside the ELD framework. These encompass the following:

1. The medico-technical classification or medical acts [7, 22]:

- non-treatment decisions such as withholding or withdrawal of life-sustaining treatment
- administering drugs or medical interventions

2. The medico-ethical classification or the life-shortening intention of the physician can be [7, 14]:

- no intention but taking into account a potentially life-shortening effect
- the potentially life-shortening effect is not the main goal but partly intended (co-intention)
- an explicit life-shortening intention.

To cover all possible decisions that could possibly influence the death of a foetus or infant, both dimensions should be taken into consideration. As a side note, intentionally ending the life of a child is illegal, meaning that in this case, the medico-ethical dimension is considered to be all the more important since no emphasis is put on the medico-technical classification specifically.

We presented this framework for validation to gynaecologists in eight individual interviews and two expert panels representing seven different hospitals. The gynaecologists were asked to give clinical examples for all possible ELD categories applied to the prenatal context, and to add more categories in case any were missing. As soon as a realistic example was given and agreed on by others, that ELD was considered possible and included in the framework (Table 1). The resulting foetal-infantile ELD framework was then thoroughly reviewed by three neonatologists.

**Table 1:**
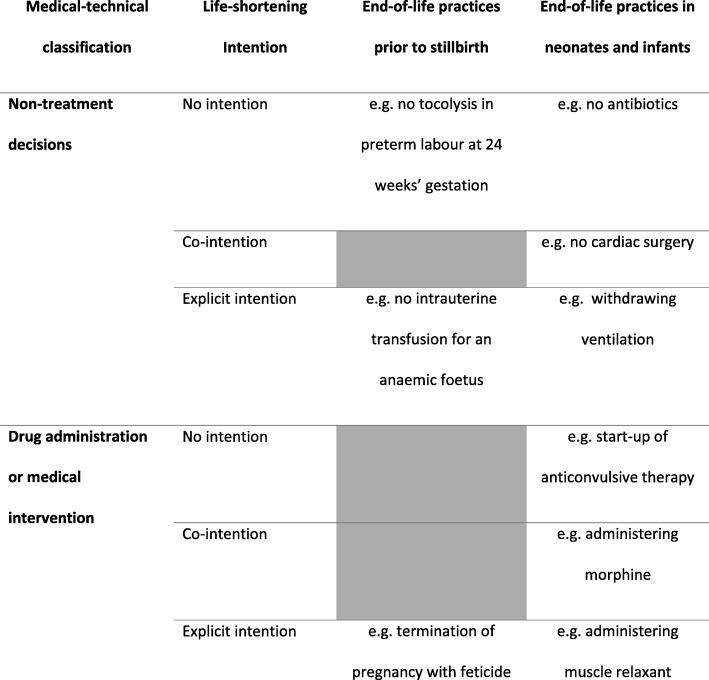
A comprehensive framework of end-of-life practices in the foetal-infantile period

Indicated in grey: not valid in the prenatal context. NTD: non-treatment decisions, APS: alleviation of pain and/or symptoms, LI: lethal interventions. This table only includes medical interventions to the child or foetus

Literature on end-of-life practices prior to stillbirth distinguishes between non-aggressive obstetric management and TOP [9, 10, 23]. Non-aggressive obstetric management (or abstinence from treatment) is the denial of interventions which are needed to sustain the life of the foetus because of a poor foetal prognosis [8–10]. TOP however, actively ends foetal life [8, 9] by preterm induction of labour either with or without feticide (administering medication to intentionally end the life of the foetus before birth) prior to the termination [24].

### Questionnaires

Based on this adjusted framework, two separate but similar questionnaires were developed for ELDs in stillborns and ELDs in neonates respectively, since both populations have their own specificities. Both questionnaires include questions about ELDs, the decision-making process, the involvement of parents in this process, the involvement of colleagues and experts, and the ELD policy of the hospital.

For neonates and infants, previously validated questionnaires that focus on end-of-life decisions in minors and neonates [14, 16, 25] were used as the basis for our questionnaire. We mainly focused on updating the terms and grammar used, term ambiguity, length of the questionnaire and comparability to the previous ELD study [14]. The resulting questionnaire was thoroughly pilot tested and validated with eight neonatologists who represented all eight Flemish NICUs, researchers in the field of end-of-life care and an ethicist.

For ELDs in stillborns a new questionnaire was developed based on previously validated questionnaires on TOP after 22 weeks [11, 12], questionnaires on ELDs in minors and neonates [14, 16, 26], and the newly developed framework for end-of-life practices in the foetal-infantile period. This questionnaire was thoroughly pilot tested and validated with eight gynaecologists, three neonatologists, researchers in the field of end-of-life care, an ethicist and a lawyer in the field of end-of-life care.

Neither questionnaire asks directly about categories of ELDs but classifies these based on a series of core questions following the two dimensions of the conceptual framework about 1) which act or omission was used (medico-technical), and 2) which life-shortening intention was associated with the act (medico-ethical). Additional questions were asked about the ways in which parents were involved in the decision-making process (parent consultation).

### Population and setting

The population includes: all stillbirths from 22 weeks of gestation or more and/or a birthweight of 500 g or higher (i.e. the internationally acknowledged limit of viability of the foetus [24, 27, 28]) and all deceased neonates and infants under the age of 1 year occurring in Flanders and Brussels where the mother is a Flemish resident. No sample is drawn; the full population is included over a data collection period of 12 months for stillbirths and 16 months for neonates and infants. The longer observation period for neonate and infant deaths was chosen because these deaths are less common than late termination stillbirths [2] and we wanted to obtain a population large enough to make reliable prevalence estimates of end-of-life practices.

Deaths to be included in the study are identified using the death certificate. Every death of a Flemish resident in Flanders and Brussels must be declared by means of a death certificate to the Flemish Agency for Care and Health of the Ministry of the Flemish Community or the Brussels Health and Social Observatory respectively. The physician, in our study most probably a neonatologist, paediatrician or gynaecologist, completes the main part of the death certificate which indicates the sex of the child, the date of birth and the date of death, medical information such as the cause of death, whether or not the child was alive at the time of birth, and the time and place of death [29]. The physician then signs the certificate and adds his or her medical registration number. The death certificate is then sent to the civil registrar of the municipality where the death took place where additional information is completed on the death certificate such as socio-demographic information about the child and its parents. Certificates are then processed by the provinces before being sent to the central administration authorities. It can take up to 3 months for death certificates to reach these administration authorities. 

### Design and procedures

**Fig. 1:**
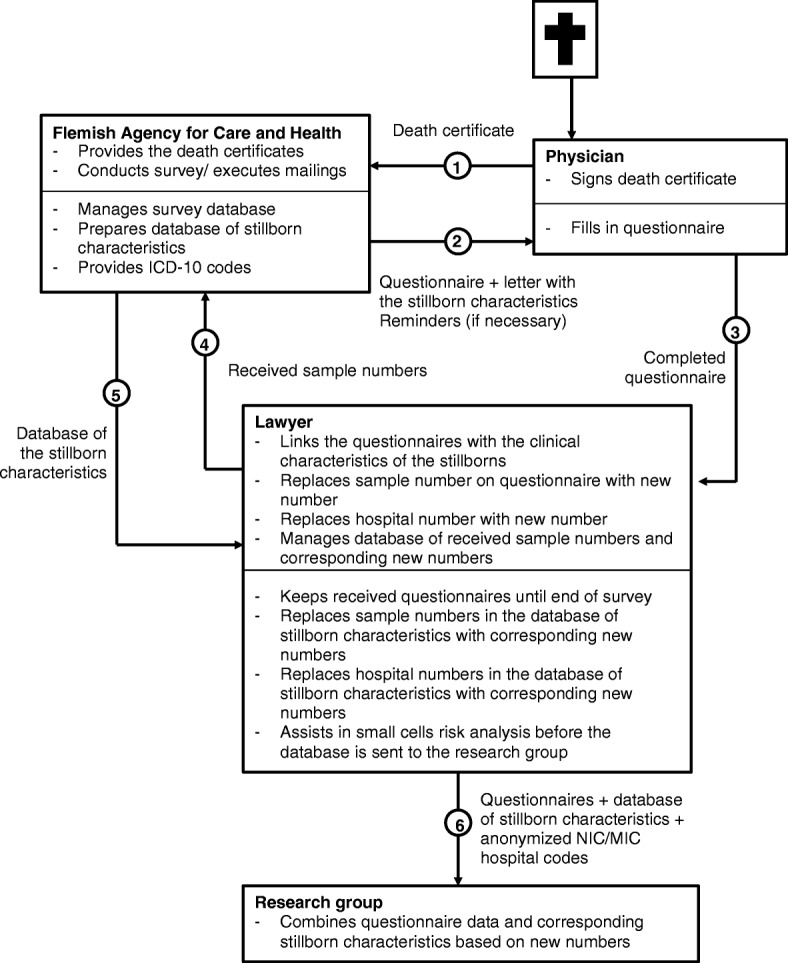
Schematic overview of the mailing and anonymity procedure

A mortality follow-back procedure is followed**,** slightly modifying well-established procedures in adults [22] and minors [26]. Modifications concern a more stringent anonymity procedure and an alternative identification procedure for stillbirths between 22 and 26 weeks. As for the anonymity procedure, ethical and legal considerations (criminal prosecution is possible for reported illegal ELDs) make it necessary to pay greater attention to the protection of confidentiality of the physician, to the privacy of the deceased, the parents and the relatives, and to the security of the data that will be obtained in the survey. By ensuring total anonymity, both the response rate and the reliability of the responses can be improved. The different stages of the survey i.e. the mailing, receiving and processing of the questionnaires will be separated and performed by four separate entities (see Fig. 1).

1. The death certificate administration authorities (namely Flemish Agency for Care and Health of the Ministry of Welfare, Health and Family of the Flemish Government) is responsible for construction and management of the mailing database and the mailing of the questionnaires. Each case is ascribed a unique coded number derived from the death certificate number. These unique numbers are used at the end of the study to link the questionnaires to the demographic and morbidity data (such as ICD-10 codes of the cause of death) of the deceased, derived from the death certificates, in a database provided by the administration authorities. An accompanying letter is included with the questionnaire providing the physician with enough patient characteristics to identify the patient. These include sex, date of (still)birth, date of death and municipality of death; for stillborns the date of death is replaced by the date of birth of the mother. When the lawyer (see below) receives the questionnaire he or she reports back to the Flemish Agency for Care and Health; all identifiable data related to the patient and the physician in question is then removed from the study database. A follow-up mailing of three reminders is performed 14, 28 and 42 days after the initial questionnaire was sent (following the Total Design Method [30]).
2. The physician identifies the deceased or stillborn child based on the patient characteristics provided, fills out the questionnaires and returns these to a lawyer using a postage paid envelope. In case the certifying physician is not the treating or attending physician he or she is given specific instructions to pass the questionnaire to the treating physician if possible.
3. The lawyer, who is bound by confidentiality, safeguards the anonymity of the questionnaires. He or she codes the participating hospital wards so that comparisons can be made, and removes any possible identifying information of hospital, physician or patient, removes the unique numbers and reports these to the administration authorities. Additionally, place of death will not be sent to the researchers in order to ensure anonymity of the participating hospitals. The lawyer links the questionnaires with the information on the database from the death certificate administration authorities, and at the end of the data collection sends the linked database to the researcher group in which all identifiers will be removed and information can no longer be traced back to the corresponding death certificate.
4. The research group receives questionnaires and ensures that both in processing and analysing the database it will not be possible to determine the identity of the patient or the physician.

**Fig. 2:**
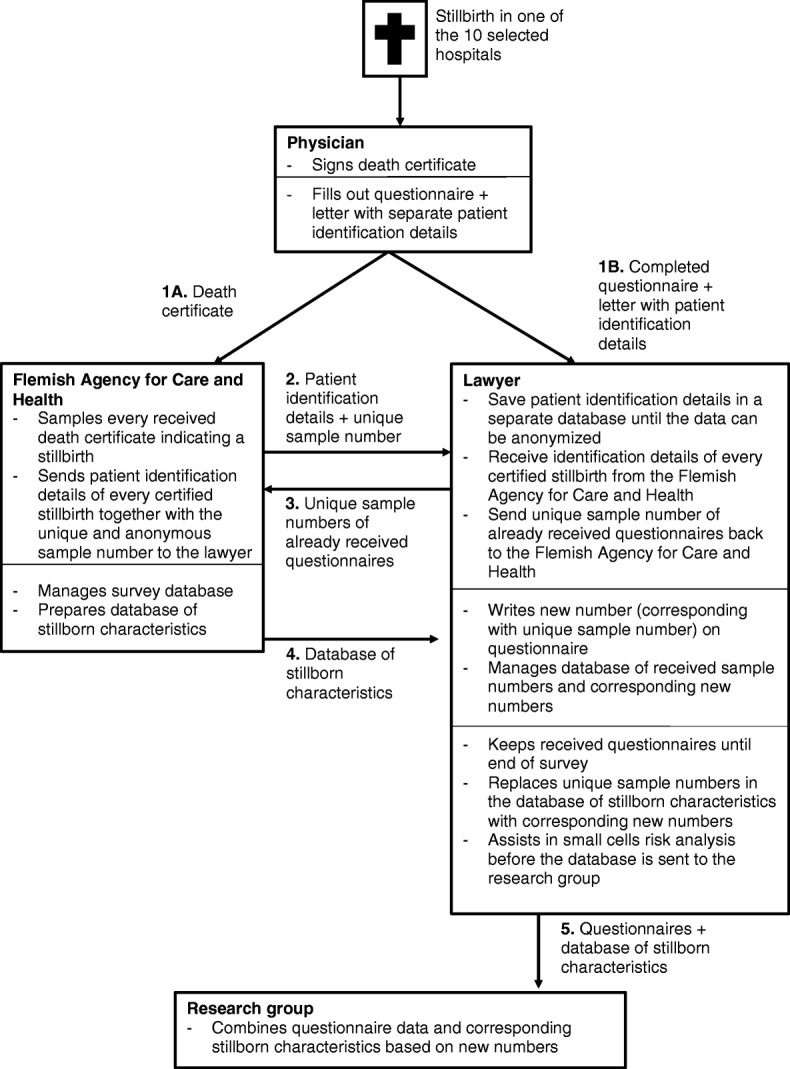
Schematic overview of the parallel procedure in the ten biggest maternity wards

An alternative identification procedure for stillbirths between 22 and 26 weeks is included because the death certificate method proves to be challenging for stillbirths in that age group. Filling in death certificates of stillbirths between 22 and 26 weeks of gestation by a physician is not mandatory, which makes the death certificates a potentially incomplete sampling framework. We provided questionnaires to the ten biggest maternity wards in Flanders and the Flemish hospitals of Brussels so that physicians can fill out this questionnaire for every stillbirth from 22 weeks of gestation onwards and/or child with a birthweight from 500 g onwards. These maternity wards were chosen based on the presence of a NICU at the hospital, because of a high birth rate and/or because they are tertiary centres for prenatal diagnostics. For each stillbirth for which a questionnaire is completed, the physician is also asked to fill out a death certificate. This makes it possible for the lawyer to link the answers in the questionnaire to the clinical and demographic characteristics of the stillborn child (for a schematic overview of this procedure, see Fig. 2). The physician sends the questionnaire, together with a separate letter containing patient identification details to the lawyer and sends the certificate to the official death certificate agency. Because the latter sends patient identification details of death certificates for stillbirths to the lawyer, the lawyer can then determine whether a questionnaire has already been received for that death and notify the Flemish agency for Care and Health via email. In this case, no questionnaires are sent by the death certificate agency. The separate letter with patient identification details is destroyed as soon as the questionnaire is linked to the corresponding death certificate. If a physician did not fill out the questionnaire available in the maternity ward but did file a death certificate, they will still receive a questionnaire through the regular postal survey.

### Improving response rates

To increase response, we follow the Total Design Method (TDM) [30]. Therefore, physicians will receive a maximum of three follow-up postal mailings. In order to further improve the response rate both in stillborns and in neonates and infants we will add an additional general follow-up. Every 3 to 4 months one of the researchers will visit all eight Flemish NICUs and all ten participating maternity wards to inquire about the course of the study. During visits, physicians will be able to ask questions, voice concerns or give general feedback. These visits are also meant to counter responder fatigue by stimulating motivation for the study duration. Furthermore, three consortium meeting will be organised to discuss the progress of the study with representatives of every NICU (one before the start, one half way through and one at the end of the study). Lastly, the study is also presented at relevant conferences and meetings.

### Ethical aspects and data protection

The sensitivity of the research population and the delicate nature of our questionnaire makes it necessary to follow a rigorous ethical approval procedure. Ethics approval was obtained from the ethics committee of the University Hospital of Ghent and additionally from the Privacy Commission (CBPL), the Sectoral Committee of Social Security and Health, and the National Council of the Order of Physicians. For our parallel procedure in the ten biggest maternity wards, we obtained ethics approval from the ethics committees of all participating hospitals.

To ensure privacy and anonymity, as well as the precautions that have already be taken by using a lawyer, we strive to ensure full data protection. The data are always password protected and stored on a protected server. The database is not replicated or shared with third parties; all copies needed for analysis are destroyed afterwards.

### Data-analysis

An SPSS 24.0 (SPSS Inc.) file is set up by the research group with a coding scheme for a certified data management company that will enter the data. The researchers will perform all data cleaning through SPSS syntax operations. Data will be analysed with descriptive statistics (valid percentages and 95% confidence intervals), bivariate and multivariate association statistics.

## Discussion

The objectives of this population study are to evaluate and monitor ELDs and their decision-making process in the foetal-infantile period including ELDs in the foetal and the neonatal period. This study design has several potential strengths as well as some limitations associated with the study population and the survey method.

### Strengths

Our study is the first to examine foetal-infantile ELDs in their entirety. The results will broaden knowledge on which medical decisions are made in cases of congenital anomalies or severe disorders from the moment of viability, regardless of whether or not the child has been born. Even though ELDs have been researched both prenatally [11, 13, 21] and in neonates [14, 16, 31], the continuity of care and the overarching decision-making process has been missed in previous studies and therefore key elements (such as whether the ELD was made prenatally but performed after the child is born) could be overlooked.

Even though there are some studies comparing late TOP practices across European countries [11, 13, 21], not much is known about the full scope of end-of-life practices before birth (including non-treatment decisions) and their decision-making process. However, non-treatment decisions such as non-aggressive obstetric management with or without explicit intention to shorten the life of the foetus can also occur. One of the strengths of our study is therefore the inclusion of all types of possible ELDs in neonates and also before birth. Furthermore, even when the child died postnatally we inquire about decisions being made prenatally and thereby provide a full overview of ELDs without prior focus on one specific ELD.

Most research on ELDs in prenatal [11, 13] and neonatal [6, 17, 20] settings is limited to single centre studies and based on reviews of medical records. Population-based studies based on officially registered death certificates, like ours, are however far more capable of obtaining robust data and reliable incidence rates since a nationwide scope ensures that the entire population is included. These could in turn lead to better understanding of current end-of-life care and detect points of improvement to benefit future parents and children with severe disorders. The only population-based study on Belgian neonatal ELDs dates back to 2000 [14] and since then, important societal changes such as questioning futile medical end-of-life care and refuting the idea of curative treatment as being necessarily beneficial could possibly have had an effect on end-of-life practice in unborn babies and neonates [32].

Aside from population specific strengths, some strengths can be attributed to the death certificate method in particular. These include international comparability, lack of patient burden and consequent attrition rates, reliability of the data, anonymity, and exclusion of possible selection bias by selecting certain physicians for the study. An overview of the strengths related to the death certificate method, which has successfully been implemented in adults [33], minors [26] and neonates [14], can be found in the research protocol of Chambaere et al. [29].

### Limitations

One of the weaknesses of the study is that the death certificate method provides a challenge in the case of stillbirth between 22 and 26 weeks of gestation because completing a death certificate is not mandatory at this age. Despite our added data collection method, we cannot guarantee 100% coverage of stillbirths. Nevertheless, the reports from the Flemish centre of Perinatal Epidemiology, which registers every birth, will be available after the study and will make it possible to estimate the number of missing cases. Furthermore, despite the additional data collection method there is also no way to ensure that physicians will always complete a death certificate (as it is not obligatory), even when they fill out the questionnaire. It is therefore possible that we will receive questionnaires which we are not able to link to a death certificate which will therefore be unusable for this study.

Delays in the processing of death certificates can reach up to 4 months before the questionnaire is sent to the physician in the first method [29]. Therefore, a recall bias cannot be excluded. However, no other registration of deaths up to the age of 1 year exists and the only other registration of all births (live and stillbirths) occurs at the Flemish centre of Perinatal Epidemiology. This consists of fewer missing cases, however, and the delay in processing these documents can be up to 1 year which would drastically decrease the reliability of the responses. Furthermore, this method of registration is due to be merged with the existing death certificate registration, making our method the most reliable for future trend research.

We include all stillbirths from 22 weeks of gestation onwards because this is internationally acknowledged to be the limit of viability of the foetus [24, 27, 28]. However, some congenital anomalies can be detected before this viability threshold so we cannot exclude an ELD having been made before the 22 weeks cut-off used in this study. Furthermore, most Flemish neonatology wards only consider viability from 24, 25 or even 26 weeks of gestation which could also have an impact on whether or not a death certificate is filled out.

### Implications for future research and practice

Regular repetition of this study in the future is needed in order to monitor and evaluate changes in end-of-life practices in the foetal-infantile group. Because this study design allows application in other countries, we recommend international comparative studies to provide us with better insight into foetal-infantile end-of-life practices and incidence rates so that international foetal and neonatal care at the end of life can be optimised.

This can eventually aid the development of obstetrical, neonatal and paediatric guidelines to support an ethical end-of-life decision-making process.

## Abbreviations

ELD: End-of-Life Decision
NICU: Neonatal intensive care unit
TDM: Total design method
TOP: Termination of pregnancy
WHO: World Health Organisation
